# Supplementary material for: Moderate-Intensity Exercise Improves Mesenteric Arterial Function in Male UC Davis Type-2 Diabetes Mellitus (UCD-T2DM) Rats: A Shift in the Relative Importance of Endothelium-Derived Relaxing Factors (EDRF)
Source: Biomedicines. 2023 Apr 8;11(4):1129. doi: 10.3390/biomedicines11041129 (PMC10136148; doi:10.3390/biomedicines11041129)
Supplement: Supplementary file 1 [file biomedicines-11-01129-s001.zip › Supplementary Figure S1, SNP Relaxation.pdf]

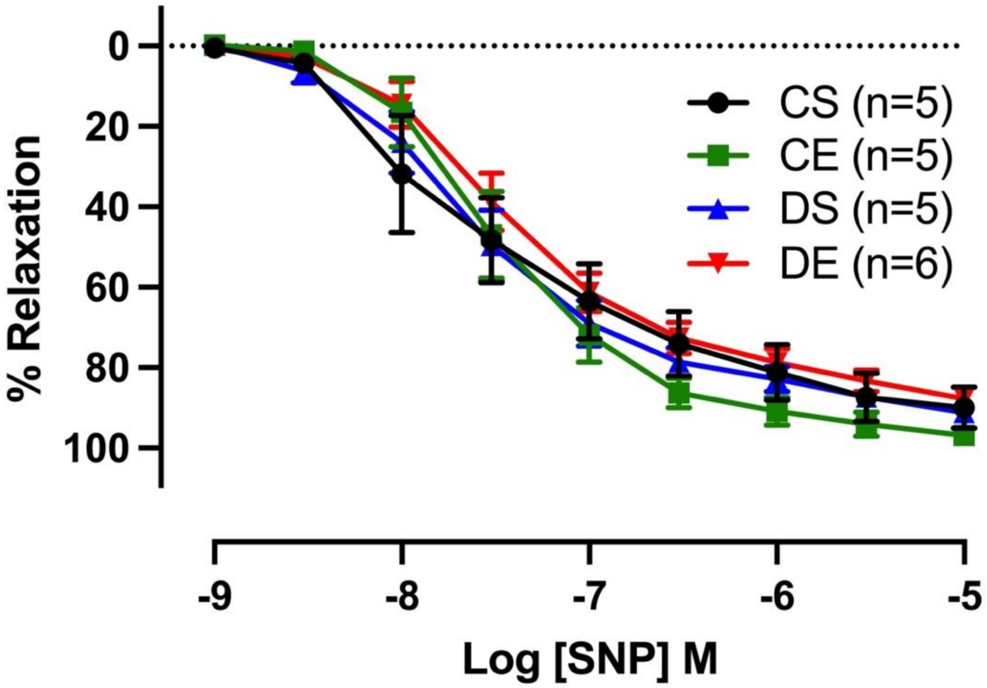

Supplementary Figure S1. Relaxation responses to cumulative concentrations of sodium nitroprusside (SNP, 10^{-9} to 10^{-5} M) in intact mesenteric arterial rings precontracted with phenylephrine (PE, 2µM) from sedentary and exercise-trained control (CS and CE) and diabetic (DS and DE) rats. Relaxation to SNP was measured in presence of indomethacin (10 µM), L-NAME (200 µM), apamin (1 µM) and TRAM-34 (1 µM). Data are expressed as mean ± SEM. n=5-6 animals per group, analyzed using two-way ANOVA with repeated measure followed by Tukey's post hoc test. NS, not significant.
