# Supplementary material for: Moderate-Intensity Exercise Improves Mesenteric Arterial Function in Male UC Davis Type-2 Diabetes Mellitus (UCD-T2DM) Rats: A Shift in the Relative Importance of Endothelium-Derived Relaxing Factors (EDRF)
Source: Biomedicines. 2023 Apr 8;11(4):1129. doi: 10.3390/biomedicines11041129 (PMC10136148; doi:10.3390/biomedicines11041129)
Supplement: Supplementary file 1 [file biomedicines-11-01129-s001.zip › Supplementary Figure S2, Full Blot Images.pdf]

A.

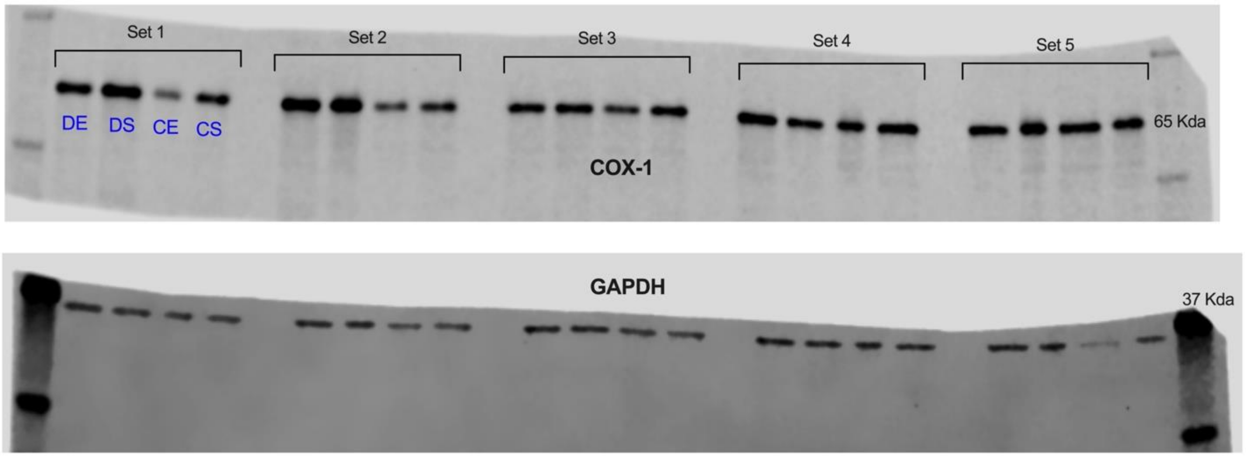
Set 1
Set 2
Set 3
Set 4
Set 5
DE DS CE CS
65 Kda
COX-1
GAPDH
37 Kda

B.

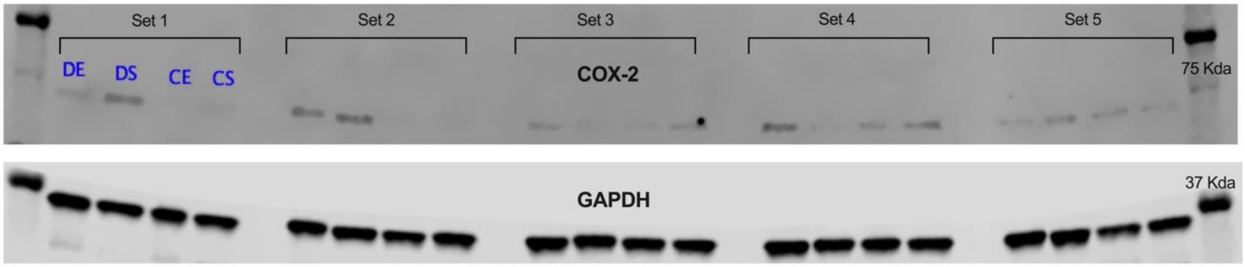
Set 1
Set 2
Set 3
Set 4
Set 5
DE DS CE CS
COX-2
75 Kda
GAPDH
37 Kda

C.

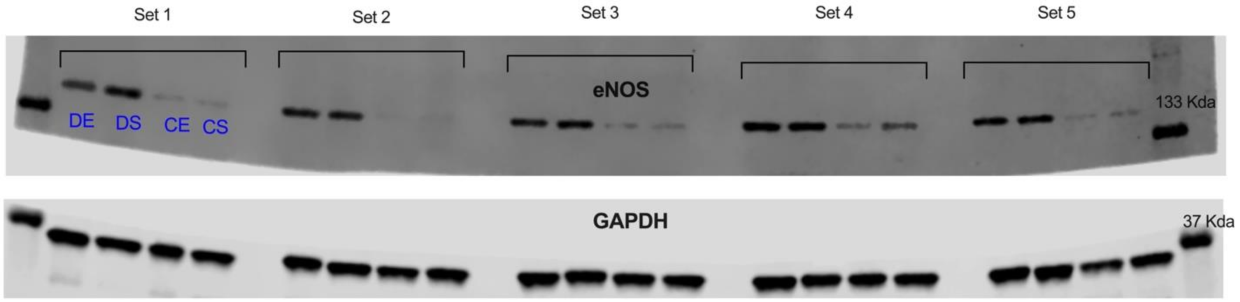
Set 1
Set 2
Set 3
Set 4
Set 5
eNOS
DE DS CE CS
133 Kda
GAPDH
37 Kda

D.

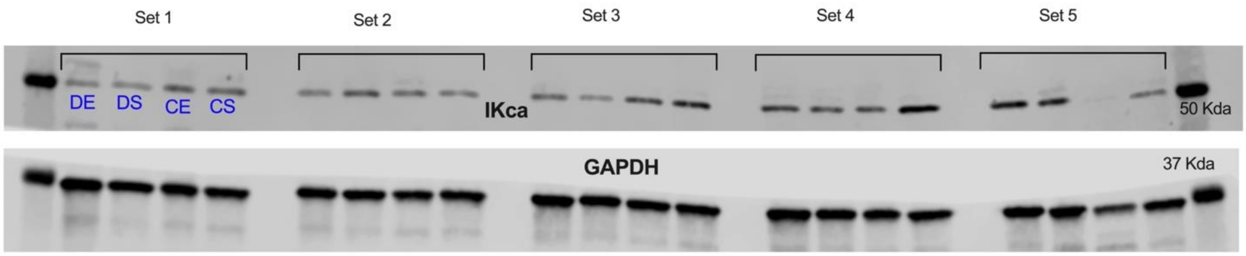
Set 1
Set 2
Set 3
Set 4
Set 5
DE DS CE CS
IKca
50 Kda
GAPDH
37 Kda

E.

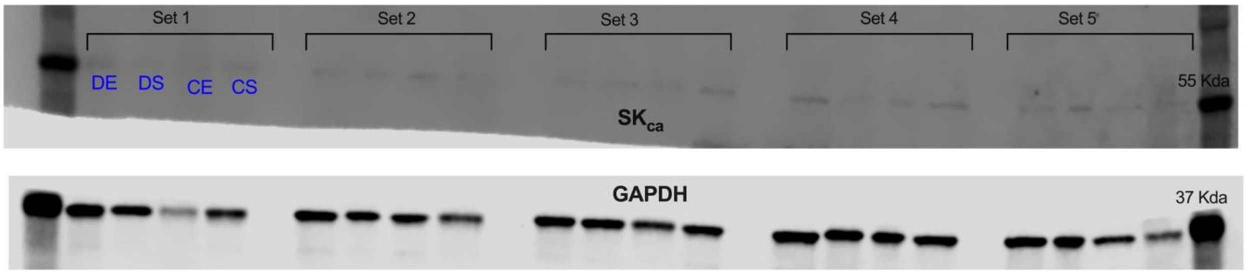

Supplementary Figure S2: Western blot analysis of (A) COX-1; (B) COX-2; (C) eNOS; (D) IK$_{Ca}$ and (E) SK$_{Ca}$ expressions in mesenteric arteries of sedentary and exercise-trained control (CS and CE) and diabetic (DS and DE) rats. Protein levels were quantified by densitometry and normalized to corresponding GAPDH (Please see **Figure 4A, 4B, 5, 6A** and **6B** in Manuscript). n=3-5 animals per group. COX-1, cyclooxygenase-1; COX-2, cyclooxygenase-2; eNOS, endothelial nitric oxide synthase; IK$_{Ca}$, intermediate conductance calcium activated potassium channel; SK$_{Ca}$, small conductance calcium activated potassium channel. Figure B and C are from the same membrane. Thus, loading control (GAPDH) is the same and GAPDH image was reused in Figure B and C. Parts of these images were used in **Figure 4A, 4B, 5, 6A** and **6B**.
