# Supplementary material for: Moderate-Intensity Exercise Improves Mesenteric Arterial Function in Male UC Davis Type-2 Diabetes Mellitus (UCD-T2DM) Rats: A Shift in the Relative Importance of Endothelium-Derived Relaxing Factors (EDRF)
Source: Biomedicines. 2023 Apr 8;11(4):1129. doi: 10.3390/biomedicines11041129 (PMC10136148; doi:10.3390/biomedicines11041129)
Supplement: Supplementary file 1 [file biomedicines-11-01129-s001.zip › Supplementary Table S1, SNP Relaxation.pdf]

Supplementary Table S1. Sensitivity (pD_2: $-logEC_{50}$), and maximum response (R_{max}) to sodium nitroprusside (SNP) in mesenteric arteries from sedentary and exercise-trained control (CS and CE) and diabetic (DS and DE) rats.

**SNP**	**n**	**pD_2($-logEC_{50}$)**	**R_{max} (%)**
CS	5	7.23±0.3	89.89±4.9
CE	5	7.38±0.2	96.88±2.1
DS	5	7.36±0.2	91.16±1.2
DE	6	7.08±0.1	87.78±2.0

Data are expressed as mean ± SEM; n=5-6 rats per group. Analyzed using one-way ANOVA followed by Tukey's post hoc test. NS, not significant.
